# Three Draft Genome Sequences of *Staphylococcus* Species Isolated from the Urine of Healthy Bovine Heifers (Gyr Breed)

**DOI:** 10.1128/MRA.00389-20

**Published:** 2020-05-21

**Authors:** Silvia Giannattasio-Ferraz, Laura Maskeri, André P. Oliveira, Edel F. Barbosa-Stancioli, Catherine Putonti

**Affiliations:** a Departamento de Microbiologia, Instituto de Ciências Biológicas, Universidade Federal Minas Gerais, Belo Horizonte, MG, Brazil; b Bioinformatics Program, Loyola University Chicago, Chicago, Illinois, USA; c Empresa de Pesquisa Agropecuária de Minas Gerais (EPAMIG), Uberaba, MG, Brazil; d Department of Biology, Loyola University Chicago, Chicago, Illinois, USA; e Department of Computer Science, Loyola University Chicago, Chicago, Illinois, USA; f Department of Microbiology and Immunology, Stritch School of Medicine, Loyola University Chicago, Maywood, Illinois, USA; Indiana University, Bloomington

## Abstract

Members of the *Staphylococcus* genus are known pathogens causing mastitis in dairy cows, which results in major economic losses. Here, we present Staphylococcus epidermidis UFMG-H7, Staphylococcus hominis UFMG-H7B, and Staphylococcus sciuri UFMG-H6, isolated from the urine of healthy purebred Gyr heifers.

## ANNOUNCEMENT

The *Staphylococcus* genus is comprised of 58 different species and 30 subspecies (1). This genus is of great relevance in animal and human health, with many staphylococci capable of pathogenicity (2). In bovines, *Staphylococcus* species are known to cause mastitis (3). S. epidermidis, S. hominis, and S. sciuri have all been reported as causing clinical and subclinical mastitis in dairy cows (4, 5). Mastitis has far-reaching financial impacts, especially in countries where livestock activities are vital to their economy. Here, we report the draft genomes of three staphylococci, namely, S. epidermidis strain UFMG-H7, S. hominis strain UFMG-H7B, and S. sciuri strain UFMG-H6, isolated from urine samples collected from 2 different dairy heifers (Gyr breed) in Brazil. These animals are from a purebred herd at the Agricultural Research Company of Minas Gerais State (EPAMIG).

Samples were collected in May 2019 and were previously approved by the Ethics Committee in Animal Experimentation of the Universidade Federal de Minas Gerais, Brazil (CEUA/UFMG; 40/2019). All of the assays were performed in accordance with relevant guidelines. For sample collection, the vulva of each animal was washed with soap and distilled water, and then a midstream urine collection was made using a 50-ml sterile tube. The material was kept frozen at −20°C for no longer than 48 hours, during which time the samples were brought back to the laboratory and processed. Aliquots of 2 ml were spun down at 10,000 × *g* for 10 minutes, and the liquid was streaked onto lysogeny broth (LB) agar plates. LB plates were incubated overnight at 37°C. Single colonies were then picked and grown in LB medium under the same conditions. This process was repeated at least 3 times to obtain pure colonies. Single colonies were picked, inoculated in LB liquid medium, and grown overnight with shaking at 37°C. From these liquid cultures, DNA was extracted using the DNeasy UltraClean microbial kit (Qiagen, Hilden, Germany) and quantified using the Qubit fluorometer. For each isolate, the 16S rRNA gene sequence was amplified (63f/1387r primers) and sequenced in order to determine the species present. DNA samples were then sent to the Microbial Genomic Sequencing Center (MiGS) at the University of Pittsburgh, where library preparation and whole-genome sequencing were performed. The DNA was fragmented using an Illumina tagmentation enzyme, and the indices were attached using PCR. The genome sequencing was conducted using the NextSeq 550 platform, producing 2 × 150-bp reads. Unless specifically noted, default parameters were used for all software tools listed. The raw reads were trimmed using Sickle v1.33 (https://github.com/najoshi/sickle) and assembled using SPAdes v3.13.0 with the “only-assembler” option for k values of 55, 77, 99, and 127 (6). All contigs were manually inspected by querying them via BLAST to the nonredundant (nr)/nucleotide database. After assembly, the genome coverage was calculated using BBMap v38.47 (https://sourceforge.net/projects/bbmap/) and annotated by the NCBI Prokaryotic Genome Annotation Pipeline (PGAP) v4.11 (7).

Draft genome assembly and annotation statistics are listed in Table 1. The isolation and characterization of these strains provide insight into *Staphylococcus* species in circulation in dairy animals.

**TABLE 1:** Draft genome assembly and annotation statistics

Parameter	Value for strain:		
	S. epidermidis UFMG-H7	S. hominis UFMG-H7B	S. sciuri UFMG-H6
Genome length (bp)	2,472,021	2,189,680	2,764,112
GC content (%)	32.36	32.36	44.36
No. of contigs	52	44	11
*N*_50_ score (bp)	140,851	100,701	522,572
Genome coverage (×)	102	118	44
No. of coding genes	2,266	2,106	2,731
No. of tRNAs	55	48	47
No. of raw read pairs	1,772,660	1,772,660	1,566,279
SRA accession no.	SRR11455648	SRR11455849	SRR11455851
WGS accession no.	JAAVMQ000000000	JAAVMU000000000	JAAVMW000000000

### Data availability.

These whole-genome sequencing (WGS) projects are available in GenBank, and raw sequence data were deposited in the SRA. Accession numbers are listed in Table 1. These genomes are deposited under BioProject accession number PRJNA615899.
